# Serum Amyloid P Is a Sialylated Glycoprotein Inhibitor of Influenza A Viruses

**DOI:** 10.1371/journal.pone.0059623

**Published:** 2013-03-27

**Authors:** Emma R. Job, Barbara Bottazzi, Brad Gilbertson, Kathryn M. Edenborough, Lorena E. Brown, Alberto Mantovani, Andrew G. Brooks, Patrick C. Reading

**Affiliations:** 1 Department of Microbiology and Immunology, University of Melbourne, Melbourne, Victoria, Australia; 2 Laboratory of Research in Immunology and Inflammation, Humanitas Clinical and Research Center, Rozzano, Milan, Italy; 3 Department of Translational Medicine, University of Milan, Milan, Italy; 4 WHO Collaborating Centre for Reference and Research on Influenza, North Melbourne, Victoria, Australia; Centre of Influenza Research, The University of Hong Kong, Hong Kong

## Abstract

Members of the pentraxin family, including PTX3 and serum amyloid P component (SAP), have been reported to play a role in innate host defence against a range of microbial pathogens, yet little is known regarding their antiviral activities. In this study, we demonstrate that human SAP binds to human influenza A virus (IAV) strains and mediates a range of antiviral activities, including inhibition of IAV-induced hemagglutination (HA), neutralization of virus infectivity and inhibition of the enzymatic activity of the viral neuraminidase (NA). Characterization of the anti-IAV activity of SAP after periodate or bacterial sialidase treatment demonstrated that α(2,6)-linked sialic acid residues on the glycosidic moiety of SAP are critical for recognition by the HA of susceptible IAV strains. Other proteins of the innate immune system, namely human surfactant protein A and porcine surfactant protein D, have been reported to express sialylated glycans which facilitate inhibition of particular IAV strains, yet the specific viral determinants for recognition of these inhibitors have not been defined. Herein, we have selected virus mutants in the presence of human SAP and identified specific residues in the receptor-binding pocket of the viral HA which are critical for recognition and therefore susceptibility to the antiviral activities of SAP. Given the widespread expression of α(2,6)-linked sialic acid in the human respiratory tract, we propose that SAP may act as an effective receptor mimic to limit IAV infection of airway epithelial cells.

## Introduction

Mammalian serum and airway fluids contain a number of soluble proteins that are known to recognize and inactivate influenza viruses. Historically, non-specific (or innate) inhibitors of influenza virus that neutralize virus infectivity and inhibit hemagglutinating activity of the virus have been classified as β or γ inhibitors based on their chemical composition and properties (reviewed by [1], [2]). β inhibitors are Ca^2+^-dependent (C-type) lectins that bind to mannose-rich glycans on the globular head of the viral hemagglutinin (HA) [3], [4]. In contrast, γ inhibitors are sialylated glycoproteins that act independently of Ca^2+^ by competing with sialylated cell-surface receptors for binding to HA.

C-type lectins of the collectin family have been implicated as a major component of innate host defense against influenza A virus (IAV) infection. Collectins express carbohydrate recognition domains (CRDs) that bind to mannose-rich glycans on the viral HA and, in some cases, to the neuraminidase (NA) [5], [6], to mediate a range of anti-IAV activities including inhibition of IAV hemagglutination and NA enzyme function, neutralization of virus infectivity, virus aggregation, increased IAV uptake by neutrophils and opsonization of virus to enhance neutrophil respiratory burst responses to IAV [7], [8], [9]. Surfactant protein (SP)-D, a collectin constitutively expressed in the lung, acts as a classical β-type inhibitor against highly glycosylated IAV [10], [11] and contributes to anti-IAV activity in human bronchoalveolar lavage (BAL) fluids [10], [12]. Mannose-binding lectin (MBL), another β inhibitor of IAV, is a serum collectin that can be detected in BAL fluids during inflammation and infection [13], [14]. The enhanced susceptibility of mice deficient in SP-D [15], [16], [17] or MBL [18] to glycosylated IAV suggests an important role for each collectin in innate host defence *in vivo*.

γ-type inhibitors were originally identified in non-immune mammalian serum and the characteristics of α2-macroglobulin, the major inhibitor of IAV in horse serum, are particularly well defined. Horse and guinea pig α2-macroglobulin express the modified sialic acid (SA) *O*-acetyl-*N*-acetyl-neuraminic acid, which resists hydrolysis by bacterial and viral sialidases and acts as a target of HA binding [19], [20]. While SP-D and MBL act as classical β inhibitors of IAV, some collectins are themselves sialylated and can mediate γ-type anti-IAV activity. For example, sialylated *N*-glycans within the CRDs of human and porcine SP-A are recognized by the HA of susceptible IAV strains [21], [22], [23]. Porcine SP-D expresses a sialylated *N*-glycan in its CRD [24] and its anti-IAV activity is the result of both Ca^2+^-dependent binding of CRD to mannose-rich glycans on the viral HA and Ca^2+^-independent binding of the viral HA to this sialylated glycan [22], [24].

Pentraxins are a phylogenetically ancient family of proteins characterized by a conserved pentameric structure comprised of five identical non-covalently linked subunits. PTX3, the prototype of the long pentraxins, and the short pentraxins SAP and C-reactive protein (CRP) have been proposed to play important roles in innate immunity and inflammation via modulation of complement activation, pathogen recognition and clearance of apoptotic cells (reviewed by [25], [26]). PTX3, SAP and CRP have been reported to bind to a variety of pathogens, however relatively little is known regarding their role in antiviral host defence. We recently demonstrated that the long pentraxin PTX3 acts as a γ-type inhibitor of IAV [27]. To gain further insight regarding the anti-IAV activities of pentraxins, we compared short pentraxins (SAP and CRP) and the long pentraxin PTX3 for their ability to bind and inhibit different strains of IAV. In contrast to previous studies indicating that SAP mediated anti-IAV activity in a manner characteristic of β-type inhibitors [28], [29], our preliminary studies demonstrated that lectin-mediated binding of SAP to IAV was not a critical determinant of its anti-IAV activity. Herein, we demonstrate that SAP expresses α(2,6)-linked sialylated glycans and acts as a classical γ inhibitor of IAV. Moreover, we have selected and characterized IAV mutants resistant to the antiviral activities of SAP, thereby defining amino acid residues critical to the interactions between SAP and susceptible IAV strains.

## Materials and Methods

### Viruses

The IAV strains used in this study were the A/PR/8/34 (PR8, H1N1), as well as HKx31 (H3N2), a high-yielding reassortant of A/Aichi/2/68 (H3N2) with PR8 that expresses the H3N2 surface glycoproteins [30]. Other viruses used in these studies were the H3N2 subtype viruses A/Memphis/1/71 (Mem/71), A/Memphis/102/72 (Mem/72), A/England/42/72 (Eng/72), A/Udorn/307/72 (Ud/72), A/Port Chalmers/1/73 (PC/73), A/Bangkok/1/79 (Bang/79), A/Beijing/353/89 (Beij/89), A/Guandong/25/93 (Guan/93), A/Wisconsin/67/2005 (Wis/05), A/Perth/16/2009 (Perth/09), A/Brisbane/10/2010 (Bris/10), as well as PR8 reassortants A/Texas/1/77 × PR8 (Tex/77, H3N2) and A/Philippines/2/82 (Phil/82, H3N2). Mem/71-Bel (H3N1) and Mem/72-Bel (H3N1) are reassortant viruses bearing the HA of Mem/71 and Mem/72, respectively, and the NA of A/Bellamy/42 (H1N1). Viruses were obtained from the World Health Organization Collaborating Centre for Reference and Research on Influenza, Melbourne, Australia. Influenza viruses were propagated in the allantoic fluid cavity of 10-day-old embryonated hens’ eggs and purified as described previously [3]. Viruses were titrated on Madin-Darby canine kidney (MDCK) cells as described [31].

Additional viruses were generated by 8-plasmid reverse genetics (RG) [32]. Reverse engineered viruses consisted of 7 genes derived from PR8 (H1N1) with the HA or NA gene from Ud/72 (PR8-Ud/72 HA and PR8-Ud/72 NA, respectively) or with the HA gene from PC/73. Viruses were rescued after 3 days and amplified in the allantoic cavity of 10-day old embryonated hens’ eggs.

IAV mutants were selected by incubation of cloned virus with purified human SAP for 30 min at 37°C prior to inoculation and amplification in eggs. Virus was harvested and screened for sensitivity to SAP by hemagglutination inhibition (HI) and selection repeated until virus had acquired resistance to SAP. Viruses were then cloned at high dilution in eggs in the absence of SAP.

### Proteins, Sera and Antibodies

SAP purified from human serum by calcium-dependent affinity chromatography on phosphoethanolamine-Sepharose [33] was purchased from Calbiochem, CA, USA. C-reactive protein (CRP) purified from human serum was purchased from Sigma Aldrich, SL, USA. Human CRP and human SAP were dialysed against 0.1M NaHCO_3_ and biotin-labelled on exposed lysine groups using 2.5 mM NHS-biotin ester (Sigma Aldrich) and subsequently dialysed against TBS containing 0.01% NaN_3_. Recombinant human PTX3 was purified from the supernatants of Chinese hamster ovary (CHO) cells stably expressing the protein and biotinylated as described [34]. Naturally occurring PTX3 was purified by immunoaffinity from human fibrosarcoma cells 8387 exposed to TNF-α (20 ng/ml) for 24 hrs. Human MBL, a kind gift from the Australian Red Cross Blood Service (Brisbane, Australia), was prepared by affinity purification from pooled human plasma using mannan agarose [35].

### Hemagglutination Titrations and HI Tests

Tests were performed in round-bottomed 96 well microtitre plates at room temperature using 1% vol/vol chicken erythrocytes in TBS (0.05 M Tris-HCl, 0.15 M NaCl [pH 7.2]). For HI tests, dilutions of PTX3, SAP or CRP were prepared in TBS supplemented with 10 mM CaCl_2_ (TBS/Ca) or 5 mM EDTA (TBS/EDTA) and 4 hemagglutinating units (HAU) of virus was added. Following 30 min incubation, chicken erythrocytes were added and the ability of pentraxin to inhibit virus-induced hemagglutination was assessed. Results are expressed as the minimum inhibitor concentration (MIC; in µg/ml) of PTX3, CRP or SAP required to fully inhibit the hemagglutinating activity of 4 HAU of virus.

### Treatment with Periodate or Bacterial Sialidases

For periodate treatment of pentraxins, 1 volume of sample was incubated with 3 volumes of 0.022 M NaIO_4_ (diluted in 0.9% w/v NaCl) for 15 min at room temperature before the NaIO_4_ was inactivated by the addition of 6 volumes of 0.22% w/v glycerol in TBS supplemented with 10 mM CaCl_2_. For mock treatment of samples, NaIO_4_ and glycerol were mixed before the addition of pentraxin.

In some experiments, pentraxins were treated with *Vibrio cholerae* Type III sialidase (Sigma Aldrich), *Clostridium perfringes* Type V sialidase (Sigma Aldrich), *Streptococcus pneumoniae* sialidase (Prozyme, CA, USA) or *Arthrobacter ureafaciens* sialidase (Roche, Germany) for 30 min at 37°C. Following treatment, bacterial sialidases were inactivated by heating at 62°C for 1 hr. For mock treatment, sialidases were heat inactivated prior to the addition of pentraxin. Note that heat inactivation of pentraxins alone did not affect HI activity against IAV (data not shown).

### Lectin Blot to Detect Sialic Acid Expression and Linkage

Fetuin, PTX3 and SAP were subjected to 12% SDS-PAGE, transferred to a PVDF membrane and SA expression were detected using the DIG Glycan Differentiation Kit (Roche Diagnostics, GmbH) according to manufacturer’s instructions. Briefly, the membrane was probed with digoxigenin (DIG)-labelled plant lectins *Maackia amurensis agglutinin* (MAA) or *Sambucus nigra agglutinin* (SNA) to detect α(2,3)- or α(2,6)-linked SA, respectively, followed by anti-DIG Fab fragments conjugated to alkaline phosphatase. Lectin binding was detected using the NBT/BCIP substrate supplied with the kit.

### Virus Neutralization Assay

Neutralization of virus infectivity was measured by fluorescent-focus reduction in monolayers of MDCK cells cultured in 96-well plates as described [27]. Briefly, dilutions of purified pentraxins prepared in TBS/Ca were mixed with standard dilution of virus, incubated at 37°C for 30 min and inoculated onto MDCK monolayers. After adsorption of virus for 1 hr at 37°C, the inoculum was removed, fresh medium was added and plates were incubated for a further 6–7 hrs. After incubation, cells were washed, fixed with 80% (v/v) acetone in water and stained for expression of newly-synthesized viral nucleoprotein (NP) using mAb MP3.10G2.IC7 (WHO Collaborating Centre for Reference and Research on Influenza, Melbourne, Australia), followed by FITC-conjugated sheep anti-mouse immunoglobulin (Silenus, Australia). The total number of fluorescent foci in four representative fields was counted and expressed as a percentage of the number of foci in the corresponding area of duplicate control wells infected with virus alone (i.e. percent of virus control).

### Enzyme-linked Immunosorbent Assays (ELISA)

Binding of PTX3, SAP or CRP to IAV or to complement component C1q was determined by ELISA. Wells of microtitre plates were coated with purified HKx31 virus (1 µg/ml) or with human C1q (10 µg/ml; Merck, Vic, Australia) in TBS, blocked for >2 hrs with 10 mg of BSA per ml and washed with TBS containing 0.05% Tween 20 (TBST). Wells were incubated with increasing concentrations of biotin-labelled PTX3, SAP or CRP in TBST containing 5 mg of BSA per ml and 5 mM CaCl_2_ (BSA_5_-TBST-Ca^2+^) or 5 mM EDTA (BSA_5_-TBST-EDTA). Binding of PTX3, SAP or CRP was detected by the addition of streptavidin-conjugated horseradish peroxidase (BD Pharmingen, NJ, USA).

To compare binding of PTX3 and SAP to wild-type or SAP-resistant (SAP^R^) viruses, plates were coated with increasing concentrations of purified viruses and probed with 2 µg/ml of biotin-labelled PTX3 or SAP. Equivalent coating levels of purified IAV preparations were confirmed by the binding of comparable levels of mAb 165, which is specific for the host-derived carbohydrate antigen characteristic of egg-grown influenza viruses [11], [36].

### NA and NA Inhibition Assays

The ability of PTX3, SAP or CRP to inhibit the activity of the viral NA was measured by an ELISA in which biotin-labelled peanut agglutinin (PNA, Pierce Biotechnology, IL, USA) was used to detect galactose residues exposed after removal of SA from fetuin (Sigma Aldrich, St Louis, MO) by the IAV NA. Dilutions of PTX3 or SAP in BSA_5_-TBS Ca^2+^ were incubated with IAV for 15 min at room temperature and transferred to fetuin-coated wells. NA activity was then determined as described [11].

### Binding of PTX3 and SAP to Influenza Virus-infected Cells

MDCK cells were infected with IAV at a multiplicity of infection (MOI) of 10 PFU/cell in serum-free media for 1 hr at 37°C, washed twice and cultured for an additional 5 hrs. To assess pentraxin binding, aliquots of 10^6^ cells were washed and incubated on ice with biotin labelled PTX3 (2 µg/ml) or SAP (10 µg/ml) in TBS containing 5 mg of BSA per ml and supplemented with either 10 mM CaCl_2_ or 5 mM EDTA, followed by streptavidin conjugated to allophycocyanin (APC). Addition of 10 µg/ml propridium iodide to each sample was used to identify viable cells prior to analysis on a FACS Calibur flow cytometer (BD Biosciences, USA).

### Desialylation and Enzymatic Resialylation of Erythrocytes

Methods for the enzymatic modification of SA residues expressed on erythrocytes have been described previously [37]. First, all viruses were adjusted to 64 HAU/well using 1% vol/vol native erythrocytes in a standard hemagglutination titration. Aliquots of 10% vol/vol chicken or human erythrocytes were desialylated with 500 mU/ml of *V. cholerae* sialidase for 1 hr at 37°C, washed twice and resialylated using 1.5 mM CMP-N-acetylneuraminic acid (NeuAc, Sigma Aldrich) and 4 mU of either β-galactoside-α(2,3)-sialyltransferase (Japan Tobacco INC, Shizuka, Japan) or β-D-galactosyl-β1,4-N-acetyl-β-D-glucosamine-α(2,6)-sialyltransferase (Merck), or with buffer alone (sham treated) at 37°C for 4 hrs. Erythrocytes were washed in TBS and the ability of IAV to bind resialylated erythrocytes was determined using a standard hemagglutination assay.

In some assays chicken erythrocytes were treated with 160 mU of *S. pneumoniae* sialidase for 1 hour at 37°C. Treated erythrocytes were subsequently tested for their ability to agglutinate viruses pre-adjusted to 64 HAU.

### Sequencing of the HA, NA and M2 Genes

RNA was extracted from infected allantoic cell pellets using RNeasy Minikit (Qiagen, Valenica, CA). Synthesis of cDNA from RNA served as a template for subsequent PCR reactions preformed with Qiagen Omniscript RT kit using 20 pmol/µL of Uni12 primer (AGC AAA AGC AGG). DNA encoding the HA, NA and M2 genes was amplified by PCR using primers as described [38]. PCR products were analysed by agarose gel electrophoresis and bands of specific size were extracted using a gel extraction kit (MO BIO, Carlsbad, CA). Sequencing was performed by Applied Genetics Diagnostics, Dept. of Pathology, The University of Melbourne. H3 numbering [39] was used to align the deduced amino acid sequences.

### Statistical Analysis

For comparison of two sets of values, a Student’s *t*-test (two-tailed, two-sample equal variance) was used. When comparing three or more sets of values, data was analyzed by One-way ANOVA followed by post-hoc analysis using Tukey’s multiple comparison test. *p* values <0.05 was considered significant.

## Results

### Human SAP and PTX3 Bind to the HKx31 Strain of IAV

**Figure 1:**
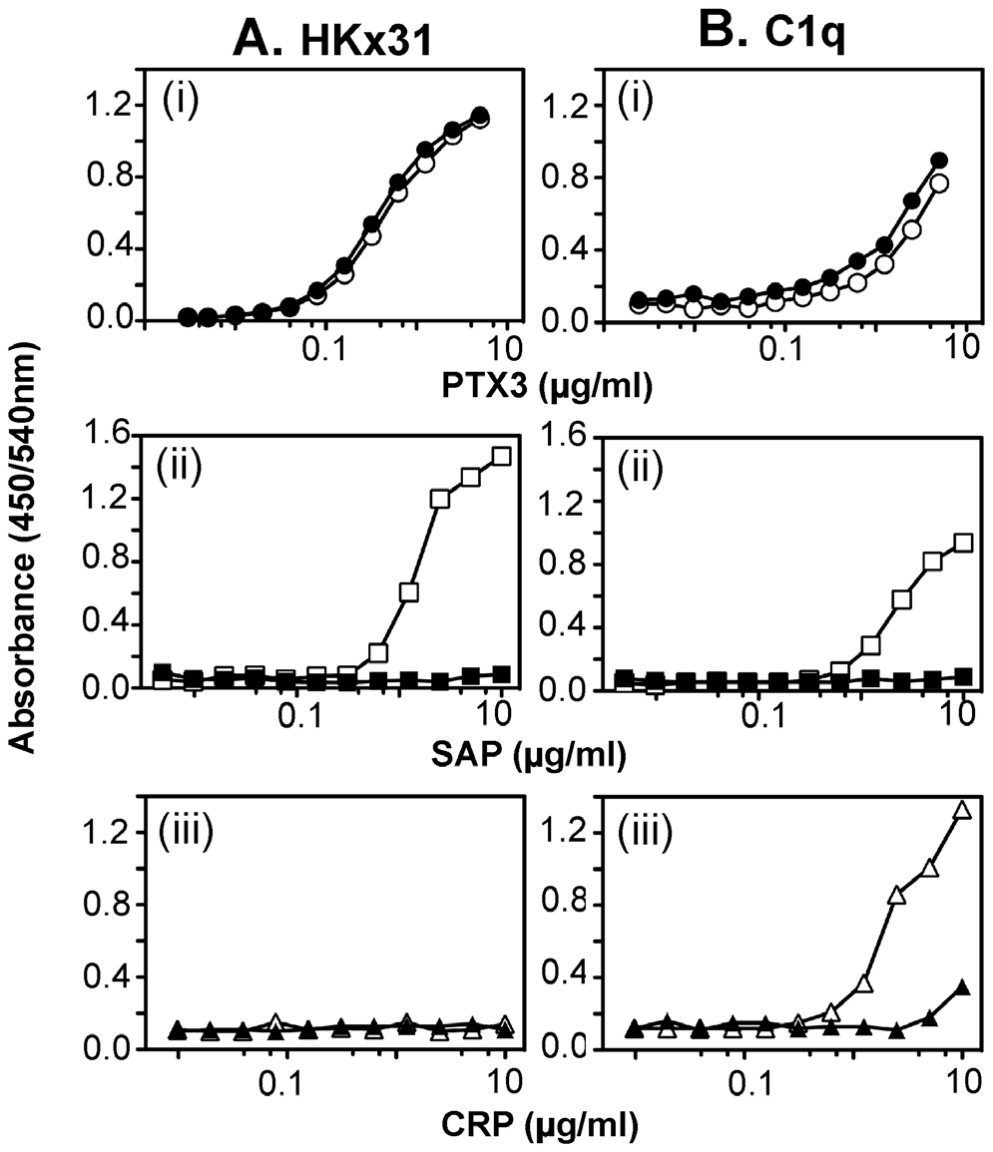
(A/B) Binding of PTX3, SAP and CRP to influenza virus HKx31 and to C1q in presence or absence of calcium. Increasing concentrations of biotin-labelled (i) PTX3 (○,•), (ii) SAP (□,▪) or (iii) CRP (△,▴) in BSA_5_-TBST supplemented with 10 mM Ca^2+^ (open symbols) or 5 mM EDTA (closed symbols) were applied to wells coated with (**A**) 1 µg/ml IAV strain HKx31, or (**B**) 5 µg/ml C1q and binding determined by ELISA. Data are representative of at least two independent experiments.

ELISA was used to assess the ability of PTX3, SAP and CRP to bind to purified IAV strain HKx31. As a control, we compared binding to complement component C1q, a known ligand for PTX3, SAP and CRP [34]. PTX3 binding to HKx31 occurred in the presence or absence of Ca^2+^ (Fig. 1A, panel (i)) whereas binding of SAP to HKx31 was Ca^2+^-dependent (Fig. 1A, panel (ii)). CRP did not bind to HKx31 when tested at concentrations of up to 10 µg/ml (Fig. 1A, panel (iii)). Consistent with previous reports [34], PTX3 bound to C1q in the presence or absence of Ca^2+^ (Fig. 1B, panel (i)) whereas binding of SAP (Fig. 1B, panel (ii)) and CRP (Fig. 1B, panel (iii)) to C1q was Ca^2+^-dependent.

### SAP does not Act as a Mannose-binding Lectin Against IAV

Next, we used HI assays to characterize the anti-IAV activity of PTX3 and SAP. Virus-induced agglutination of chicken erythrocytes was inhibited by PTX3 and SAP (Table 1), but not by CRP at concentrations up to 10 µg/ml (data not shown). Consistent with previous studies, the inhibitory activity of SAP was abrogated by chelation of Ca^2+^ with EDTA [28], whereas PTX3 mediated HI activity in the presence or absence of Ca^2+^ [27] (Table 1).

**Table 1:** HI activity of PTX3, SAP and MBL against IAV.

Diluent	MIC (µg/ml)^a^		
	PTX3	SAP	MBL
+10 mM CaCl_2_	0.625	0.312	0.312
+5 mM EDTA	0.625	>10*	>10*
+10 mM CaCl_2_+100 mM D-mannose	0.625	0.312	>10*
+10 mM CaCl_2_+100 mM L-rhamnose	0.625	0.312	0.312

a MIC are representative of 2 or more independent experiments using HKx31.

* ≥4-fold difference in MIC relative to results in +10 mM CaCl_2_.

Previous studies reported that inclusion of the sugar D-mannose inhibited the anti-IAV activities of SAP [28], suggesting that SAP bound to mannose-rich glycans expressed on IAV HA/NA glycoproteins. In our hands, SAP was not inhibited by 100 mM D-mannose or by 100 mM L-rhamnose, whereas mannose blocked the anti-IAV activity of the collectin MBL (Table 1). SAP was equally potent against H3 subtype strains Mem/71-Bel or Phil/82 as it was against β inhibitor-resistant mutants of each strain which lack mannose-rich glycans from the head of HA [3], [40] (Table S1, Exp 1, including bovine serum as a known β inhibitor of IAV). Furthermore, treatment of HKx31 with periodate to oxidize the carbohydrate moieties of the HA/NA glycoproteins did not affect its sensitivity to inhibition by SAP (Table S1, Exp. 2). Together, these findings demonstrate SAP does not act as a classical β-type inhibitor of IAV and that its anti-IAV activity is not mediated via recognition of mannose-rich glycans on IAV HA/NA.

### Sialic Acid on SAP Plays a Critical Role in its Ability to Mediate Antiviral Activity against IAV

**Figure 2:**
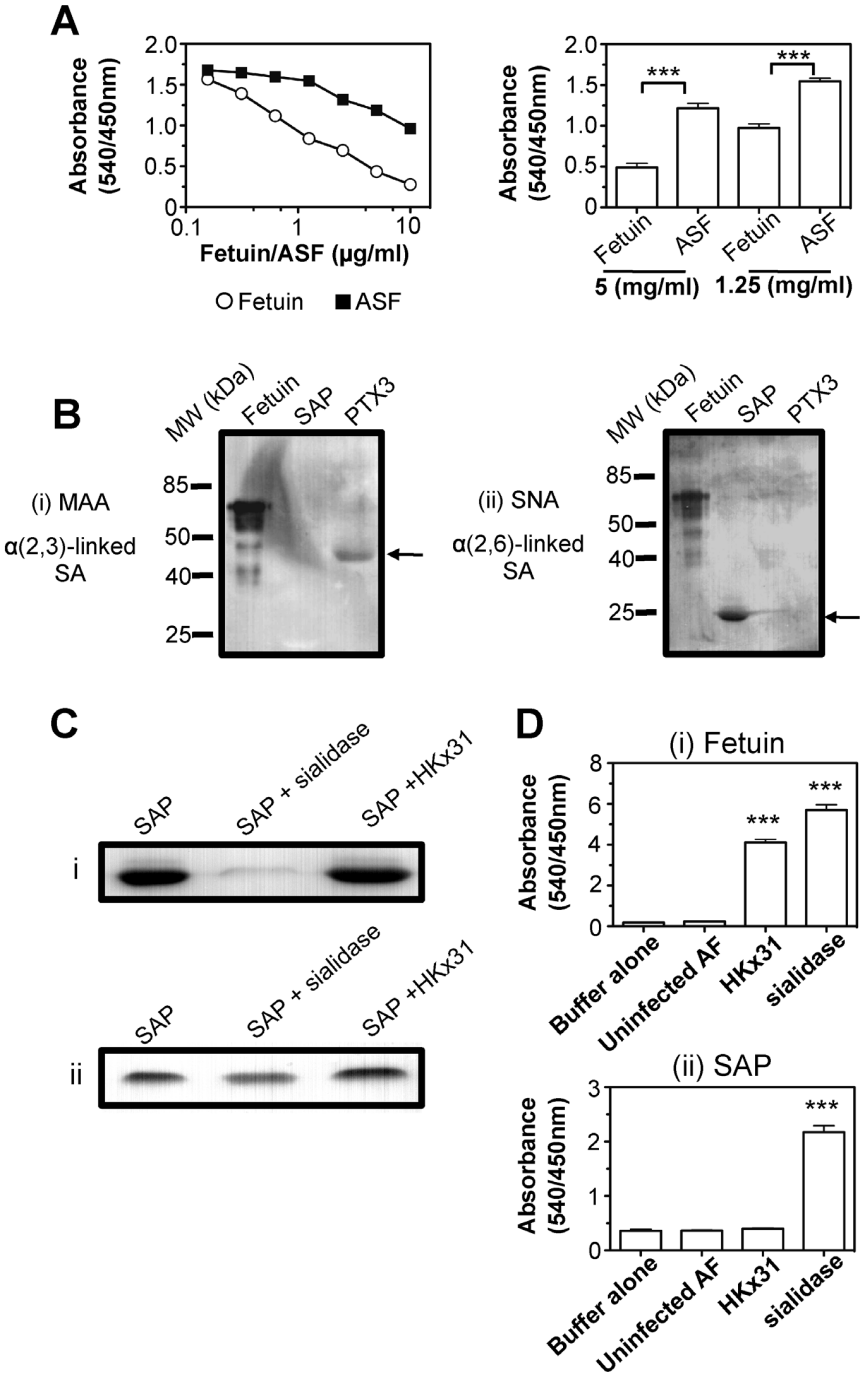
Sialic acids expressed by SAP are resistant to hydrolysis by the viral NA. (**A**) Wells coated with 1 µg/ml of HKx31 were incubated with increasing concentrations of fetuin or asialofetuin (ASF) for 1 hr before addition of 5 µg/ml biotin-labelled SAP. Binding of biotin-labelled SAP to HKx31 was then determined by ELISA. All incubations and washes were performed at 4°C using chilled buffers to inhibit the enzymatic activity of the viral NA. Data show a single titration (left panel) as well as triplicate wells at a specific concentration of fetuin/ASF (right panel). The absorbance of biotin-labelled SAP binding in the absence of fetuin/ASF was 1.558 (±0.105). ***p<0.001, Student’s t-test for equivalent concentrations of fetuin compared to ASF. (**B**) Purified PTX3 and SAP were subjected to 12% SDS-PAGE under reducing conditions followed by lectin blot using DIG-conjugated MAA (panel i) or SNA (panel ii) for detection of α(2,3)- or α(2,6)-linked SA, respectively. Fetuin was included as a positive control for each SA linkage. (**C**) SAP (2.5 µg) was incubated for 1 hr at 37°C with sialidase from *V. cholerae* (25 mU), HKx31 (1/20 dilution of allantoic fluid) or with buffer alone and then analysed by SDS-PAGE and DIG-lectin blot using DIG-SNA (panel i). Ponceau S stain confirmed similar amounts of SAP were transferred to the PVDF membrane prior to the lectin blot (panel ii). (**D**) ELISA plates coated with (i) fetuin (20 µg/ml), or (ii) SAP (40 µg/ml) were incubated with HKx31 (1/10 dilution of allanotic fluid) or sialidase from *V. cholerae* (0.5 mU) for 30 min at 37°C and removal of SA was determined using NA assay as described in Material and Methods. Uninfected allantoic fluid (AF) was included as a negative control. Data shows the mean absorbance (±1 SD) of triplicate wells and is representative of at least two independent experiments. ***p<0.001, One-way ANOVA compared to uninfected allantoic fluid control or buffer alone control.

To determine if SAP acts as a sialylated inhibitor of IAV, we first examined the ability of fetuin, a highly sialylated glycoprotein, to block binding of SAP to IAV. As seen in Fig. 2A, fetuin inhibited binding of biotin-labelled SAP to HKx31 more effectively than did an equivalent concentration of asialofetuin (ASF).

Next, we used periodate treatment to oxidize the glycans expressed by SAP and PTX3. Compared to mock-treated controls, periodate treatment abrogated the ability of PTX3 and SAP to mediate HI activity against HKx31 (Table 2, Exp. 1). We then compared the ability of SAP and PTX3 to mediate anti-IAV activity following treatment with different bacterial sialidases (Table 2, Exp. 2). Although sialidases from *V. cholerae* and *C. perfringes* show a general preference for α(2,3)- over α(2,6)-linked SA, differences in structure and/or core oligosaccharides expressed by the target protein can modulate specificity and cleavage rate [41]. Treatment of SAP and PTX3 with *V. cholerae* sialidase abrogated anti-IAV activity whereas sialidase from *C. perfringes* inhibited only SAP. Moreover, *A. ureafaciens* sialidase which preferentially recognises α(2,6)- over α(2,3/8)-linked SA abrogated the anti-IAV activity of SAP, but not PTX3, whereas the α(2,3)-specific sialidase from *S. pneumoniae* inhibited PTX3, but not SAP. Lectin blot using DIG-conjugated lectins (Fig. 2B) confirmed that PTX3 expressed α(2,3)-, but not α(2,6)-linked SA [42], whereas SAP expressed α(2,6)-, but not α(2,3)-linked SA [42], [43].

**Table 2:** Sialic acids expressed by PTX3 and SAP are critical for antiviral activity against influenza virus.

Exp.	Diluent	Treatment of pentraxin	MIC (µg/ml)^a^	
			PTX3	SAP
**1** b	+10 mM CaCl_2_	None	0.625	0.312
	+10 mM CaCl_2_	Periodate (0.022 M)	>5.0*	>5.0*
	+10 mM CaCl_2_	Mock - Periodate	1.25	1.25
**2** b	+10 mM CaCl_2_	*V. cholerae* (50 mU)	>10.0*	>10.0*
	+10 mM CaCl_2_	Mock - *V. cholerae*	0.156	0.312
	+10 mM CaCl_2_	*C. perfringens* (50 mU)	0.156	>10.0*
	+10 mM CaCl_2_	Mock - *C. perfringens*	0.312	0.312
	+10 mM CaCl_2_	*A. ureafaciens* (5 mU)	1.25	10.0*
	+10 mM CaCl_2_	Mock - *A. ureafaciens*	0.625	0.625
	+10 mM CaCl_2_	*S. pneumoniae* (10 mU)	>10.0*	0.312
	+10 mM CaCl_2_	Mock - *S. pneumoniae*	0.312	0.312

a MIC are representative of 2 or more independent experiments using HKx31.

b Treatment with periodate or sialidase and the preparation of appropriate mock-treated controls are described in Material and Methods.

* ≥4-fold difference in MIC relative to appropriate mock-treated control.

An important feature of γ-inhibitors is their ability to resist hydrolysis by the IAV NA. We used two approaches to confirm that SA on SAP was resistant to the viral NA. First, SAP was incubated with allantoic fluid containing HKx31 or with sialidase from *V. cholerae* for 1 hr at 37°C and then DIG-conjugated SNA was used to detect α(2,6)-linked SA on SAP (Fig. 2Ci). Treatment of SAP with bacterial sialidase markedly reduced SA expression whereas incubation with HKx31 did not. A Ponceau S stain confirmed similar amounts of SAP were compared in each sample (Fig. 2Cii). Second, ELISA plates coated with the SA-rich glycoprotein fetuin or with SAP were incubated with *V. cholerae* sialidase or with allantoic fluid containing HKx31 at 37°C for 30 mins. After this time, biotin-labelled PNA was used to detect galactose residues exposed by removal of SA. As seen in Fig. 2D, HKx31 could cleave SA residues from fetuin (panel i), but not from SAP (panel ii) whereas *V. cholerae* sialidase cleaved SA from both fetuin and SAP. Together these data confirm that SA on SAP is resistant to hydrolysis by the viral NA.

### Correlation between Viral HA Receptor Specificity for Sialic Acid and Sensitivity to PTX3 or SAP

**Figure 3:**
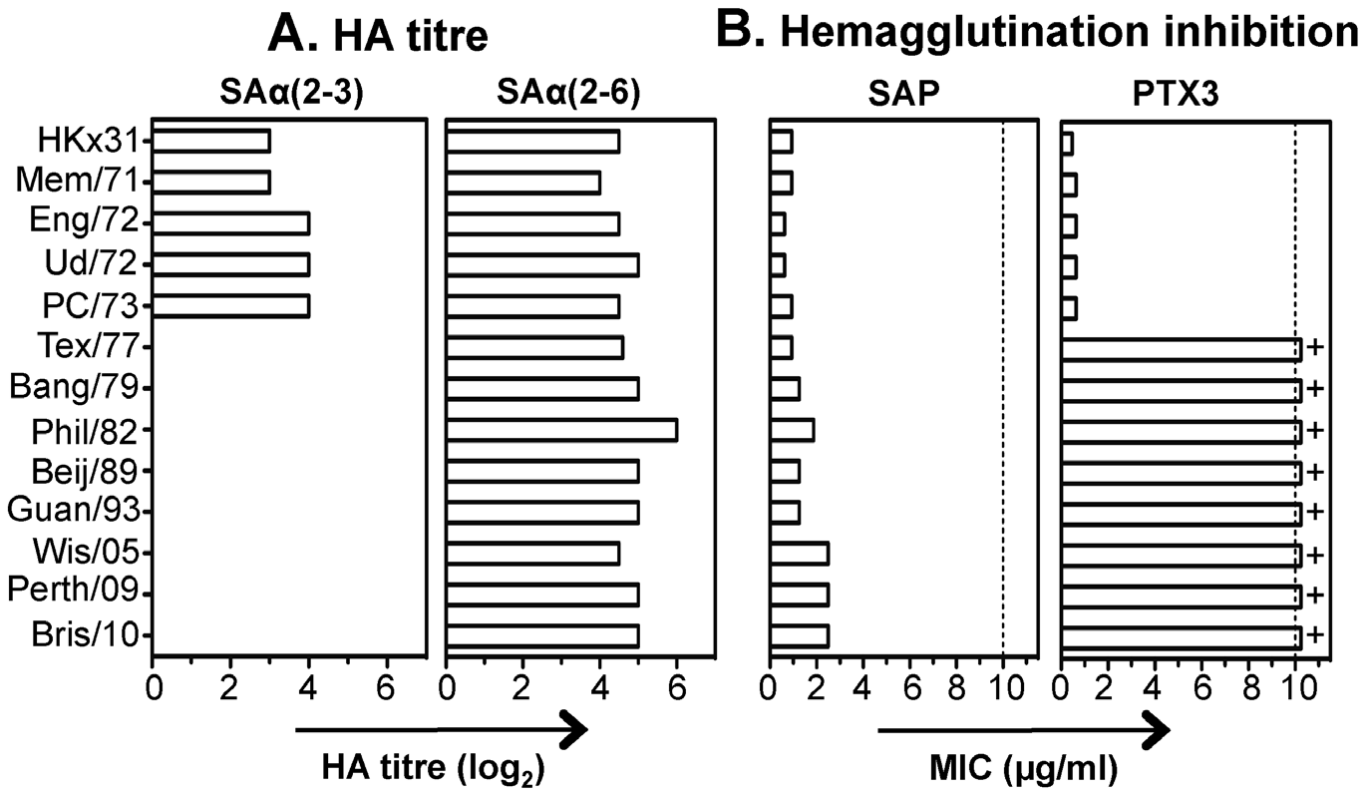
HA receptor specificity of H3 subtype IAV strains correlates with sensitivity to SAP and PTX3. (**A**) Human erythrocytes were desialylated and subsequently resialylated with α(2,3)- or α(2,6)-sialyltransferases and used in hemagglutination assays. All viruses were adjusted to 64 HAU on native erythrocytes and showed no hemagglutinating activity (<1 HAU) against desialylated erythrocytes (data not shown). (**B**) Inhibition of HA activity of H3 subtype IAV by pentraxins. SAP or PTX3 were diluted in TBS containing 10 mM Ca^2+^ and used in a standard HI assay. Bars indicate the minimum concentration of SAP or PTX3 required to inhibit 4 HAU of each virus tested. Dashed line represents the highest concentration of inhibitor tested (10 µg/ml). ‘+’ indicates a value of >10 µg/ml. Data are representative of 2 or more independent experiments.

As the glycosidic moieties on SAP and PTX3 express distinct SA linkages, we hypothesized that SAP would differ from PTX3 in its spectrum of anti-IAV activity. We compared a range of H3 subtype IAV for receptor specificity (Fig. 3A) and for sensitivity to PTX3 and SAP (Fig. 3B). Consistent with previous reports [44], H3 viruses isolated between 1968–1973 agglutinated erythrocytes expressing either α(2,3)- or α(2,6)-linked SA whereas viruses isolated after 1973 agglutinated only those that expressed α(2,6)-linked SA (Fig. 3A). Early (1968–1973), but not later (post-1975) H3 strains were inhibited by PTX3 (Fig. 3B), consistent with the ability of early strains to bind α(2,3)-linked SA and expression of α(2,3)-linked SA by PTX3. Note that naturally occurring PTX3 purified from TNF-α stimulated human fibrosarcoma cells also expressed only α(2,3)-linked SA ([42] and personal communication, Dr. Antonio Inforzato, Istituto Clinico Humanitas, Milan, Italy) and showed similar activity against H3N2 subtype viruses to that of recombinant PTX3 expressed by CHO cells (data not shown). In contrast, all H3 strains tested recognized α(2,6)-linked SA (Fig. 3A) and all were sensitive to inhibition by SAP (Fig. 3B). Note that CRP did not mediate HI against any of the H3 IAV strains tested (MIC >10 µg/ml).

### SAP Mediates a Range of Antiviral Activity against Influenza Viruses *in vitro*

**Figure 4:**
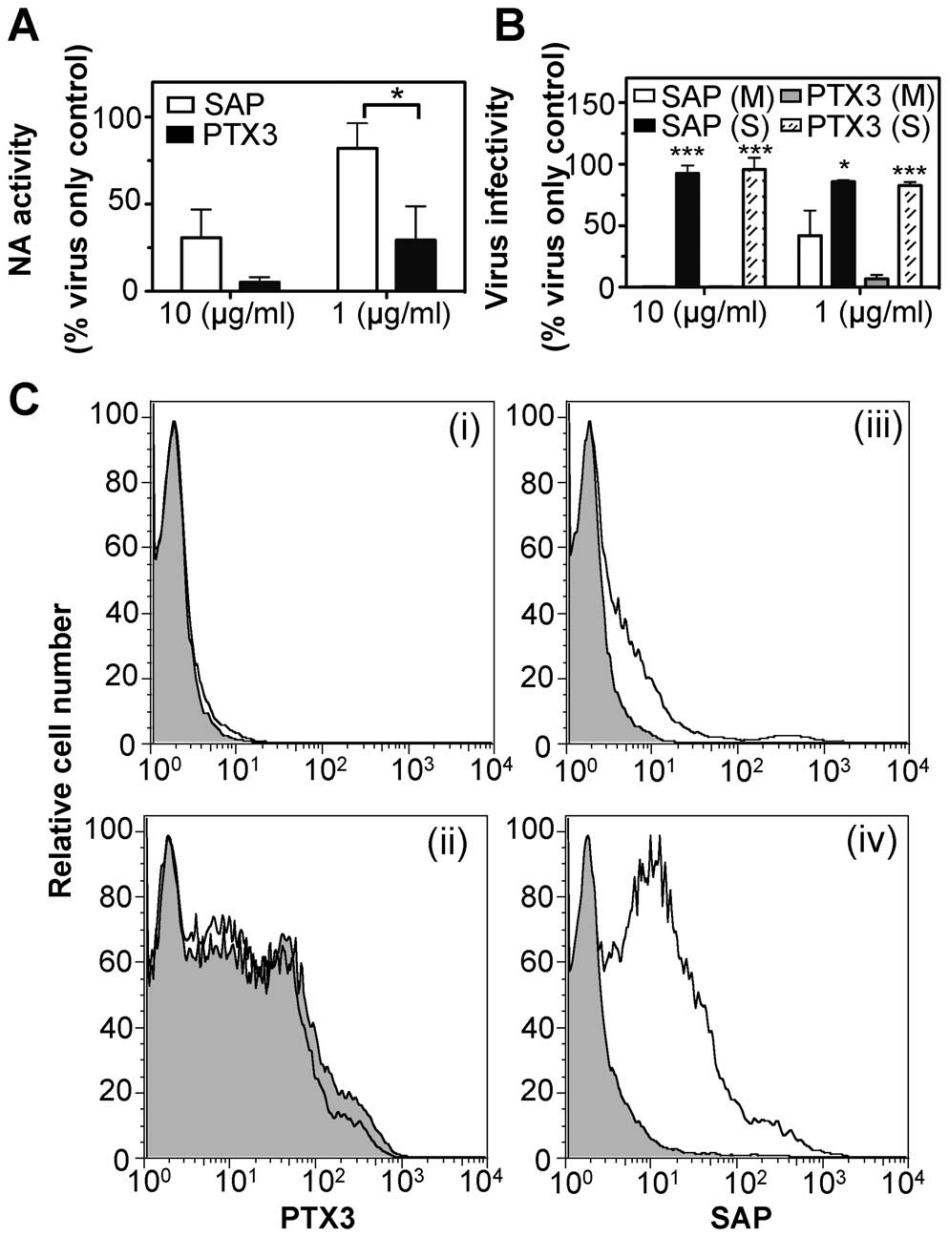
Antiviral activities of SAP and PTX3 against IAV and IAV-infected cells. (**A**) Inhibition of NA activity by SAP and PTX3. A dilution of HKx31 was mixed with a final concentration of 10 or 1 µg/ml of SAP (white bars) or PTX3 (black bars) in BSA_5_-TBS-Ca^2+^ and assayed for NA activity as described in Materials and Methods. Results are expressed as the percent of NA activity compared to a virus only control. Data shown are the mean (±1 SD) from three independent experiments. *p<0.05, Students *t*-test, significant difference in levels of NA activity in the presence of SAP compared to an equivalent concentration of PTX3. (**B**) Neutralization of IAV by SAP and PTX3. Pentraxins were treated with sialidase from *Vibrio cholerae* (50 mU) in TBS containing 10 mM Ca^2+^ or mock-treated (M) in buffer alone for 30 min at 37°C, and then heated to 56°C for 30 min to inactivate sialidase. Sialidase- (S) or mock- (M) treated pentraxins were mixed with a dilution of HKx31 to give a final concentration of 10 or 1 µg/ml of PTX3 or SAP, and the amount of infectious virus remaining determined by fluorescent-focus reduction assay. Results are expressed as a percent of the number of fluorescent foci in the virus only control. Data show the mean (±1 SD) from three independent experiments. *p<0.05, ***p<0.001, Student’s *t*-test, significant difference between sialidase-treated sample compared to appropriate mock-treated control. (**C**) Binding of SAP and PTX3 to IAV-infected MDCK cells. Binding of SAP and PTX3 to uninfected (i, iii) or HKx31-infected MDCK cells (ii, iv) was determined at 6 hrs post-infection. Aliquots of 10^6^ cells were incubated with biotin-labelled SAP (10 µg/ml) or PTX3 (2 µg/ml) in BSA_5_-TBS-Ca^2+^ (white histograms) or BSA_5_-TBS-EDTA (grey histograms). Binding of biotin-labelled pentraxins was determined by flow-cytometry. Histograms are representative of two independent experiments.

Pre-incubation of influenza viruses with proteins of the innate immune system can inhibit the enzymatic activity of the viral NA [11], [27], [45] and neutralize virus infectivity [5], [11], [27], [28]. Both SAP and PTX3 inhibited NA activity (Fig. 4A) and neutralized virus infectivity (Fig. 4B) in a dose-dependent manner although PTX3 displayed more potent antiviral activity in each assay. Consistent with HI data shown in Table 2, pre-treatment of PTX3 and SAP with *V. cholerae* sialidase abrogated their neutralizing activity against IAV (Fig. 4B). During natural infection, IAV would be derived from growth in human respiratory epithelial cells. We confirmed that SAP inhibited the ability of IAV to infect human airway epithelial cell lines, including BEAS-2B and A549 (data not shown). Moreover, IAV strains propagated in BEAS-2B cells were as sensitive to neutralization by SAP as their egg grown counterparts (Figure S1), indicating that human SAP mediates anti-IAV activity against virus grown in cells of the homologous system.

Prior to release of newly synthesized virions, HA and NA are expressed at the surface of IAV-infected cells and represent potential targets for recognition by innate pattern recognition receptors (PRRs). Consistent with our previous report [27], PTX3 bound to IAV-infected, but not uninfected cells, in the presence or absence of Ca^2+^ (Fig. 4C, panels (i) and (ii)). SAP also bound IAV-infected, but not uninfected cells, and binding of SAP to IAV-infected cells was Ca^2+^ dependent (Fig. 4C, panels (iii) and (iv)). Together, these data demonstrate the potential of SAP to mediate anti-IAV activities against both free virions and against virus-infected cells.

### SAP Binds to the HA of Influenza Viruses to Mediate Antiviral Activity

**Figure 5:**
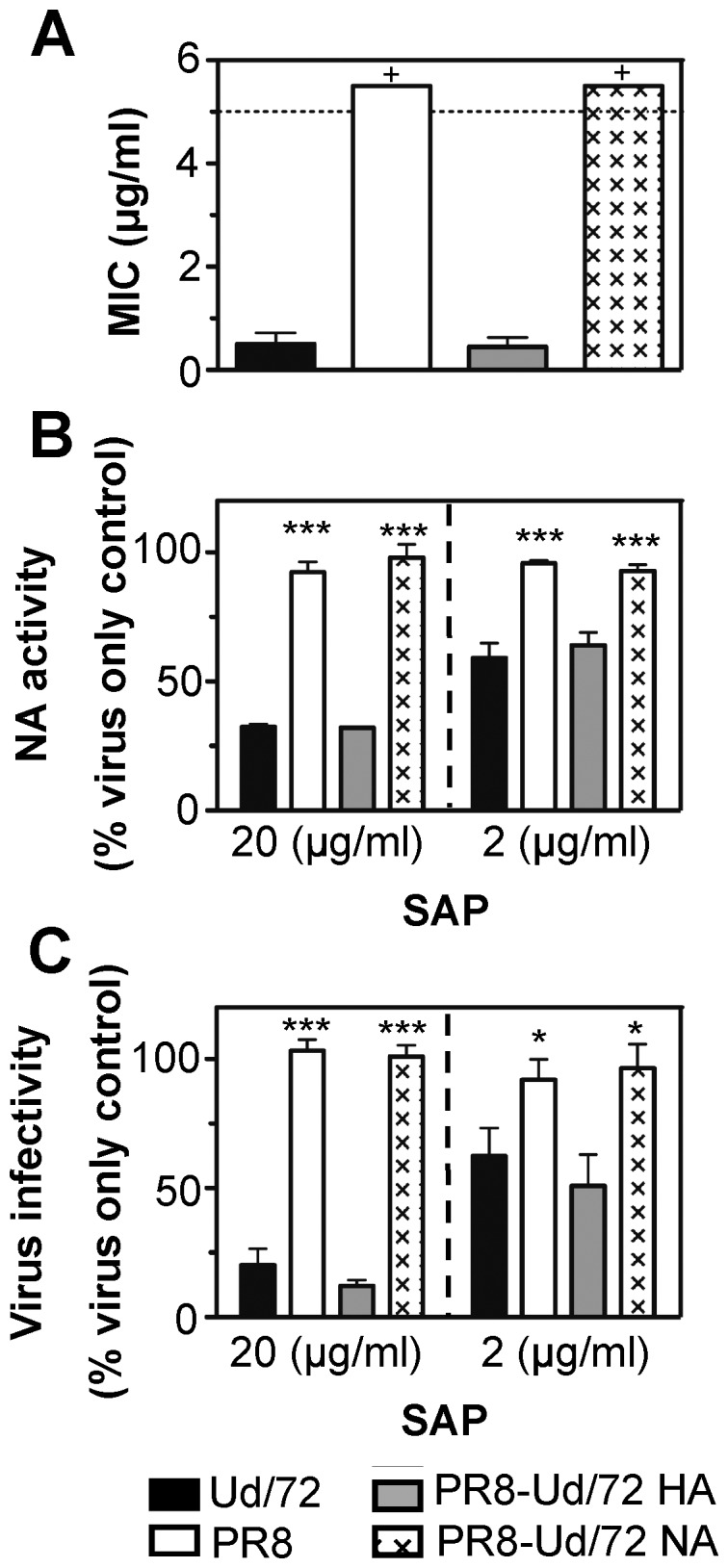
SAP binds to the HA glycoprotein of IAV to mediate its antiviral activities. SAP was tested for its ability to mediate a range of anti-IAV activities against Ud/72, PR8, PR8-Ud/72 HA or PR8-Ud/72 NA. (**A**) Inhibition of virus-induced hemagglutination. Dilutions of SAP in TBS/Ca were tested for anti-IAV activity in standard HI assays. MIC values are the mean (±1 SD) concentration of SAP required to inhibit 4 HAU of virus. Dotted line indicates the maximum concentrated tested (5 µg/ml) and ‘+’ indicates a MIC value >5 µg/ml. (**B**) Inhibition of viral NA activity. Dilutions of virus were mixed with SAP (at a final concentration of 20 µg/ml or 2 µg/ml and assayed for NA activity. Results are expressed as the percent of NA activity compared to a virus only control. (**C**) Virus neutralisation. A dilution of each virus was mixed with SAP diluted in TBS/Ca to a final concentration of 20 µg/ml or 2 µg/ml and then incubated for 30 min at 37°C. The amount of infectious virus remaining was then determined by fluorescent-focus reduction assay. Results are expressed as a percent of the number of fluorescent foci in the virus only control. Data shown in A, B and C are the mean of three independent experiments (±1 SD). *p<0.05, ***p<0.001, One-way ANOVA, significantly different to viruses expressing Ud/72 HA (i.e. Ud/72 and PR8-Ud/72 HA).

SAP inhibited activities associated with IAV HA and NA. To determine if SAP recognized one or both IAV glycoproteins we used 8-plasmid reverse genetics (RG) to generate wild-type SAP-sensitive (SAP^S^) Ud/72 (H3N2) and SAP-resistant (SAP^R^) PR8 (H1N1), as well as reverse engineered 7∶1 viruses with a PR8 backbone that expressed either HA or NA of Ud/72 (PR8-Ud/72 HA or PR8-Ud/72 NA, respectively). Each virus was tested for (i) sensitivity to HI (Fig. 5A), (ii) inhibition of the enzymatic activity of NA (Fig. 5B), and (iii) neutralization of virus infectivity by SAP (Fig. 5C). Viruses bearing the HA of Ud/72 (i.e. Ud/72 and PR8-Ud/72 HA) were more sensitive to HI and to neutralization by SAP than viruses bearing the HA of PR8 (i.e. PR8 and PR8-Ud/72 NA). Sensitivity of the viral NA to inhibition by SAP also correlated with expression of the Ud/72 HA, suggesting that SAP binds the HA of susceptible virus strains where it may sterically block the active site on neighbouring NA molecules; a similar mechanism has been proposed for inhibition of NA activity by members of the collectin superfamily [11], [45].

### Selection and Characterization of SAP-resistant Mutants of H3 Subtype IAV

A mutant of H3 subtype strain Mem/71-Bel selected for resistance to bovine serum (a rich source of the β inhibitor conglutinin) was generated which had acquired a Thr to Asn substitution at position 167 (Thr_167_→Asn), resulting in loss of a potential glycosylation site from the tip of the HA spike [3]. In contrast, a horse serum-resistant mutant of Mem/71-Bel was characterized by a single amino acid substitution Leu_226_→Gln in HA, which changed the receptor-binding specificity from preferential recognition of α(2,6)-linked SA to α(2,3)-linked SA [46], [47]. To gain further insight into the mechanisms by which SAP inhibited IAV, we selected a SAP^R^ mutant of Mem/71-Bel. Sequencing genes encoding HA and NA of wild-type (WT) and SAP^R^ viruses identified a single amino acid substitution (Leu_226_→Gln) in the receptor-binding pocket of HA associated with resistance to SAP. This substitution was identical to that associated with resistance of H3 subtype IAV to horse serum and the HS^R^ mutant of Mem/71-Bel which was also resistant to SAP (data not shown).

**Figure 6:**
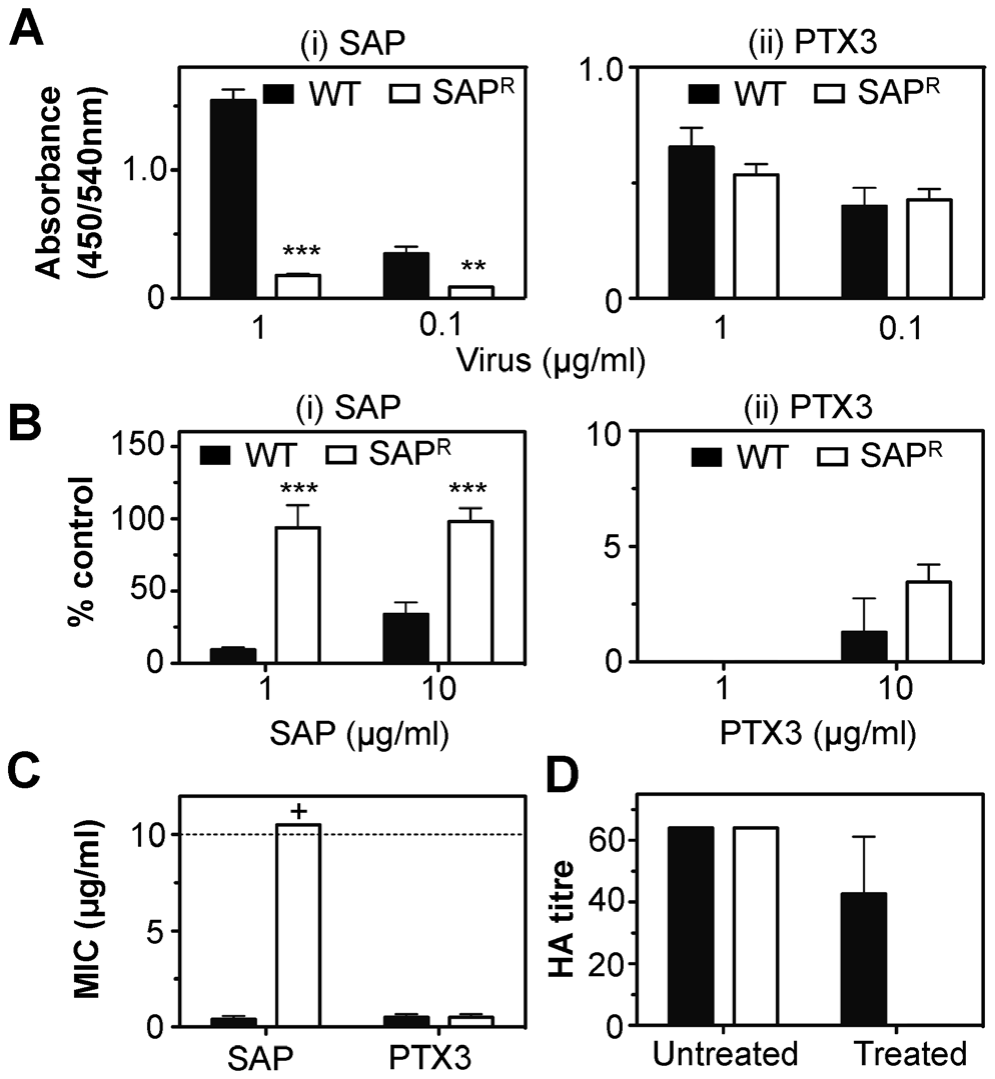
SAP^R^ mutants of H3 subtype IAV remain sensitive to inhibition by PTX3. (**A**) Binding of SAP or PTX3 to purified Mem/71-Bel WT or Mem/71-Bel SAP^R^. Biotin-labelled (i) SAP (2 µg/ml) and (ii) PTX3 (2 µg/ml) diluted in BSA_5_-TBST-Ca^2+^ were applied to wells coated with 1 µg/ml or 0.1 µg/ml (as indicated) of purified virus and binding of SAP or PTX3 was determined by ELISA. Equivalent coating levels of purified viruses was confirmed using mAb 165 as described in Materials and Methods (data not shown). Data represent the mean of three independent experiments (±1 SD). **p<0.01, ***p<0.001, Student’s t-test, comparing SAP^R^ virus to WT virus for each pentraxin. (**B**) Neutralization of Mem/71-Bel WT and Mem/71-Bel SAP^R^ by SAP or PTX3. Viruses were mixed with an equal volume of PTX3 or SAP prepared in TBS/Ca (to a final concentration of 10 or 1 µg/ml of each pentraxin, as indicated), incubated for 30 min at 37°C and the amount of infectious virus remaining was determined by fluorescent-focus reduction assay. Results are expressed as a percent of the number of fluorescent foci in the virus only control and represent the mean (±1 SD) from three independent experiments. ***p<0.001, Student’s t-test, comparing SAP^R^ to WT virus for each pentraxin. (**C**) Inhibition of virus-induced hemagglutination by SAP and PTX3. Dilutions of PTX3 or SAP in TBS +10 mM Ca^2+^ were tested for their ability to inhibit hemagglutination by Mem/71-Bel WT (black bars) or Mem/71-Bel SAP^R^ (white bars). Results show the minimum inhibitory concentration (MIC) of SAP or PTX3 required to inhibit 4 HAU of virus. Dashed line represents the highest concentration of inhibitor tested (10 µg/ml); values above this line designated >10 µg/ml are indicated by a ‘+’. Data represent mean (±1 SD) from three independent experiments. (**D**) Mem/71-Bel SAP^R^ virus does not agglutinate erythrocytes treated with *S. pneumoniae* sialidase. Chicken erythrocytes were treated with 160 mU of *S. pneumoniae* sialidase for 1 hour at 37°C. Mem/71-Bel (WT, black bars) or Mem/71-Bel SAP^R^ (SAP^R^, white bars) were adjusted to 64 HAU on untreated chicken erythrocytes and assayed for their ability to agglutinate sialidase-treated erythrocytes. Data represent mean (±1 SD) from three independent experiments.

We compared SAP and PTX3 for their ability to mediate antiviral activity against WT and Mem/71-Bel SAP^R^. SAP bound purified WT, but not Mem/71-Bel SAP^R^ (Fig. 6Ai), whereas PTX3 bound both viruses (Fig. 6Aii). Moreover, Mem/71-Bel-SAP^R^ was resistant to neutralization (Fig. 6B), HI (Fig. 6C) and inhibition of NA enzymatic activity (data not shown) by SAP whereas PTX3 showed similar activity against WT and Mem/71-Bel SAP^R^ in each assay. Consistent with previous reports characterizing horse serum-resistant mutants of H3 subtype IAV [46], the Leu_226_→Gln mutation in HA associated with resistance to human SAP altered the receptor-binding specificity of the viral HA. Mem/71-SAP^R^ failed to agglutinate erythrocytes treated with the α(2,3)-specific sialidase from *S. pneumoniae* (Fig. 6D), consistent with reduced avidity for α(2,6)-linked SA.

**Figure 7:**
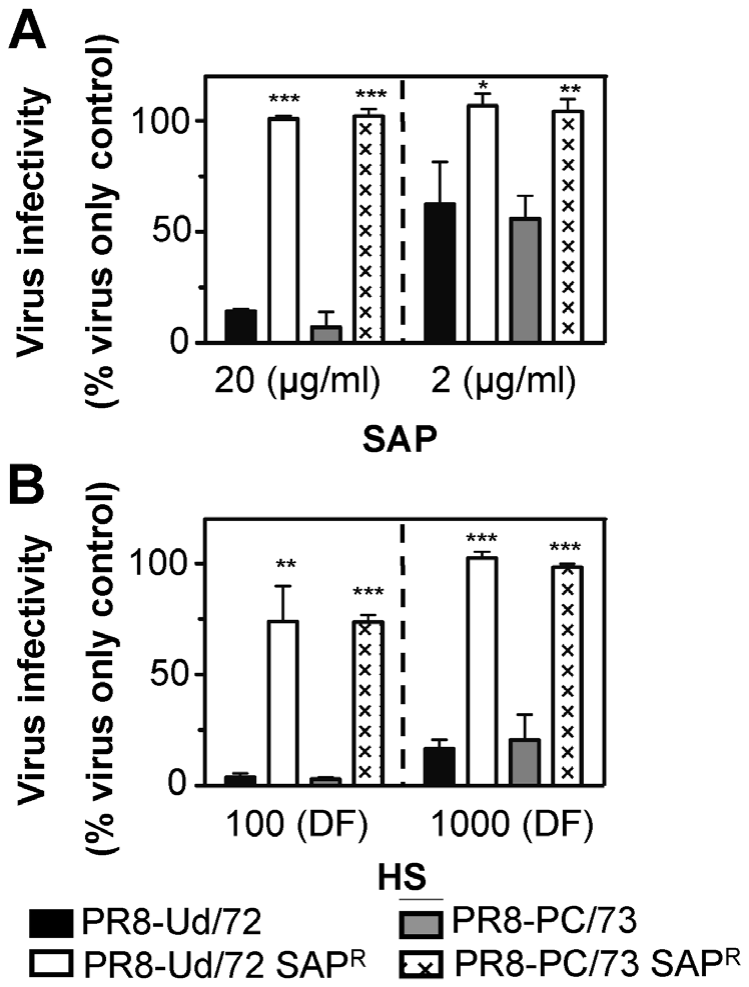
Neutralization of RG viruses by SAP or horse serum. Dilutions of WT or SAP^R^ PR8-PC/73 HA and PR8-Ud/72 HA viruses in TBS were incubated for 30 min at 37°C with an equivalent volume of (**A**) SAP, at a final concentration of 20 µg/ml or 2 µg/ml in TBS/Ca, or (**B**) horse serum (HS), at a final dilution factor (DF) of 100 or 1000 in TBS/Ca, and the amount of infectious virus remaining was determined by fluorescent-focus reduction assay. Data are expressed as a percent of the number of fluorescent foci in the virus only control. Data show the mean of three independent experiments (±1 SD). *p<0.05, **p<0.01, ***p<0.001, Student’s *t*-test, comparing each SAP^R^ virus to its appropriate WT control.

We next generated RG reassortants expressing 7 genes from PR8 in conjunction with the HA from H3 subtype strains Ud/72 or PC/73. Thus, the background of each virus was genetically identical in order to control for mutations in other viral genes that might contribute to SAP resistance. SAP^R^ mutants of each RG virus contained the L226Q substitution in HA and no other mutations. All SAP^R^ mutants failed to agglutinate erythrocytes resialylated to express α(2,6)-linked SA (data not shown) and all were resistant to neutralization by SAP (Fig. 7A) and by horse serum (Fig. 7B). Furthermore, a RG virus engineered to express 7 genes from PR8 in conjunction with Ud/72 HA containing the L226Q substitution was resistant to SAP (data not shown). Published sequences from H3 subtype viruses (1968–1990) indicate that Leu_226_ is highly conserved in the HA of human H3 subtype viruses isolated prior to 1990 (Table S2). Only A/Queensland/7/1970 was found to contain Leu_226_ and this strain was not available for further analysis.

### SAP Enhances the Neutralizing Activity of β-type Inhibitors against WT, but not SAP^R^ IAV

**Figure 8:**
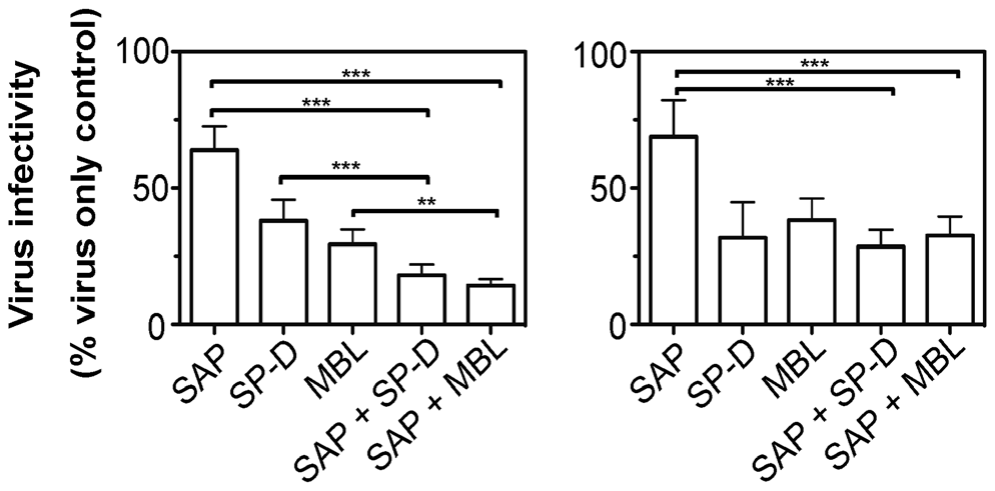
SAP enhances the neutralizing activity of SP-D and MBL against WT but not SAP^R^ Mem/71-Bel. Dilutions of (i) SAP, SP-D and MBL were prepared in TBS supplemented with 10 mM Ca^2+^ alone, or (ii) SP-D and MBL were prepared in TBS supplemented with 10 mM Ca^2+^ and 0.60 µg/ml SAP, and incubated at 37°C for 15 mins to allow for interactions between different proteins. After this time, an equivalent volume of WT or SAP^R^ Mem71-Bel was added and incubated at 37°C for 30 mins and the amount of infectious virus remaining was determined by fluorescent-focus reduction assay. Final concentrations after incubation with virus were SAP alone (0.6 µg/ml), SP-D alone (0.3 µg/ml), MBL alone (2.5 µg/ml), SAP+SP-D (0.6 µg/ml +0.3 µg/ml) and SAP+MBL (0.6 µg/ml +2.5 µg/ml final). Neutralization data for WT (left panel) or SAP^R^ Mem/71-Bel (right panel) are expressed as the mean (±1 SD) percent of the number of fluorescent foci in each virus only control and are pooled data from two independent experiments, each performed in triplicate. **p<0.01, ***p<0.001, One-way ANOVA, Tukey’s post hoc.

Human airway fluids contain a complex mixture of innate immune proteins capable of mediating anti-IAV activity. Previous studies have indicated that particular proteins which inhibit IAV can augment or interfere with the anti-IAV activities of other innate proteins [12], [48], [49]. Therefore, we investigated whether SAP modulated the neutralizing activity of SP-D or MBL, two β-type inhibitors of IAV that are present in airway fluids during IAV infection [10], [11], [12], [18]. Using a concentration of SAP which weakly inhibited IAV, we demonstrate cooperative neutralizing activity against WT Mem71-Bel when SAP was added to preparations of either SP-D or MBL (Fig. 8, left panel). Of interest, this effect was not observed against SAP^R^ Mem/71-Bel (Fig. 8, right panel), indicating that the cooperative effects of SAP likely reflect interactions between SAP and virus, rather than SAP binding to SP-D/MBL, or to the MDCK cells.

## Discussion

Previous studies reported that SAP binds in a Ca^2+^-dependent manner to mannose-rich glycans on the HA of susceptible strains to mediate anti-IAV activity [28], [29]. Our data confirm Ca^2+^-dependent binding of SAP to IAV, however antiviral activity was unaffected (i) in the presence of mannose, (ii) by oxidation of glycans on the virion, or (iii) against mutants of H3 subtype IAV resistant to β inhibitors and lacking mannose-rich glycans from the tip of the HA spike. Instead, the sialylated glycan expressed by SAP was identified as the target recognized by susceptible IAV strains. SAP expressed α(2,6)-linked SA and inhibited a broad range of human H3N2 viruses, consistent with HA receptor preference for α(2,6)-linked SA. In contrast, PTX3 expressed α(2,3)-linked SA and was active only against H3N2 strains circulating between 1968–1975 which exhibited dual preference for α(2,3)- and α(2,6)-linked SA. Furthermore, H3 subtype mutants selected for resistance to human SAP showed reduced avidity for α(2,6)-linked SA and remained sensitive to PTX3.

The distinct linkages and positioning of glycans expressed by SAP (α(2,6)-linked SA at Asn_32_) and PTX3 (α(2,3)-linked SA at Asn_220_) [34], [43] are likely to contribute to the distinct patterns of reactivity seen against different strains of IAV (Fig. 3). In contrast to the single SA linkages expressed by SAP and PTX3, human SP-A expressed both α(2,3)- and α(2,6)-linked SAs [23] and showed similar potency against α(2,3)-specific (PR8 (H1N1)) or α(2,6)-specific (Phil/82 (H3N2)) IAV strains [23]. Porcine SP-D functions as a unique natural inhibitor of IAV in that it mediates simultaneous β-type activity (i.e. CRD-mediated binding to mannose-rich glycans on IAV HA/NA) and γ-type activity (i.e. the α(2,6)-rich glycan in its CRD is recognized by the HA of α(2,6)-specific virus strains), resulting in enhanced anti-IAV activity as well as a broader range of susceptible IAV strains [22], [24], [50].

Equine α2-macroglobulin and human SAP express α(2,6)-linked SA [19], [43]. It has long been established that preferential binding of IAV strains to α(2,6)-linked SA correlates with sensitivity to α2-macroglobulin in horse serum [20], [46] and horse serum-resistant mutants of early H3 subtype viruses were characterized by the L226Q mutation in HA [46]. We report the same mutation to be associated with resistance to human SAP. Published sequences from H3 subtype viruses (1968–1990) indicate that Leu_226_ is highly conserved in the HA of human H3 subtype viruses (Table S2), consistent with their preferred specificity for α(2,6)-linked SA (Fig. 3, [37], [51]). Given the widespread expression of α(2,6)-linked SA in the human respiratory tract, SAP could act as an effective receptor mimic and inhibit IAV infection of respiratory epithelial cells. However, the emergence and spread of SAP-resistant variants in humans is unlikely as reduced recognition of α(2,6)-linked SA would compromise virus infection and amplification in human airways. The conservation of Leu_226_ in circulating H3 subtype IAV strains (Table S2) argues that SAP may represent an important component of innate immunity that IAV strains cannot readily evade.

It is well established that the 4-*O*-acetyl-*N*-neuraminic acid expressed by equine α2-macroglobulin resists hydrolysis by bacterial sialidases and IAV NA and can neutralize IAV infectivity [19], [20]. However, the anti-IAV activity of SAP was sensitive to sialidases from *V. cholerae*, *C. perfringes* and *A. ureafaciens*, but not the α(2,3)-specific sialidase from *S. pneumoniae* (Table 2), indicating that the sialylated moiety expressed by SAP is likely to be distinct from that expressed by equine α2-macroglobulin. Moreover, while sialidase from *V. cholerae* removed SA from SAP, the HKx31 NA did not (Fig. 2C/D) and therefore SAP mediated potent neutralizing activity against this strain of IAV (Fig. 4B). Variation in the core oligosaccharide and tertiary structure of glycoproteins can influence accessibility and/or rate of cleavage by different sialidases [41], [52]. Moreover, SAs are subject to a remarkable number of modifications, generating a family of more than 50 structurally distinct molecules (reviewed in [53], [54]). Structural diversity is generated primarily by modifications of hydroxyl groups at C4, C7, C8 and C9 by acetate, lactate, sulphate or phosphate esters and modified SAs tend to be resistant to microbial sialidases. SAP has been reported to express a single sialylated di-antennary glycan [43], however detailed biochemical analyses are required to determine the particular SA modifications expressed by human SAP that account for its resistance to hydrolysis by IAV NA.

Unlike SAP and PTX3, the related pentraxin CRP is generally not glycosylated [55], consistent with our inability to detect binding or HI activity against any of the IAV strains tested. Glycosylated molecular variants of human CRP are, however, induced in some pathological conditions [56] and show distinct patterns of binding to serum glycoproteins when compared to the non-glycosylated protein [57]. Glycosylated variants of CRP differed not only in SA content but also in linkage specificity to sub-terminal sugars and variants expressing α(2,3)- or α(2,6)-linked SA were induced in response to different disease conditions [57]. CRP used in our studies was purified from human serum, however it will be of interest to determine if CRP is also present in airway fluids and, if so, to analyze its glycosylation status during IAV infection and/or pulmonary inflammation. The sialylated di-antennary glycan expressed by SAP does not display the microheterogeneity characteristic of many mammalian glycoproteins [43] and although its function is not fully understood it has been proposed to be involved in pentamer-pentamer associations [58]. In contrast, PTX3 preparations from different cell types show heterogeneity in the relative amounts of bi, tri and tetrantennary glycans and removal of SA from PTX3 potentiates its ability to bind certain ligands such as C1q [42].

Binding of HA to SA ligands occurs independently of Ca^2+^, yet we report the antiviral activities of SAP to be Ca^2+^-dependent. Previous studies demonstrate that PTX3 does not contain the specific co-ordination sites for Ca^2+^ that are characteristic of CRP and SAP [34], consistent with our findings that Ca^2+^ was required for binding of CRP and SAP, but not PTX3, to C1q (Fig. 1A). In the absence of Ca^2+^, SAP forms decamers composed of two pentameric subunits whereas SAP isolated in the presence of Ca^2+^ has been reported to auto-aggregate [59], [60]. In addition, significant conformational differences have been reported in the secondary structure of SAP in the presence or absence of Ca^2+^ [61]. Such changes are likely to alter the spatial arrangement of sialylated oligosaccharides and/or accessibility of the glycosidic moiety to the viral HA, consistent with our findings that the ability of SAP to bind (Fig. 1B) and mediate HI activity (Table 1) against IAV was abrogated in the presence of EDTA.

SAP is expressed in hepatocytes and present in the serum of healthy adults at levels of 30–50 µg/ml [62], [63]. In mice, SAP enters the airways during acute respiratory infection [64], however to our knowledge there are no reports quantifying SAP levels in bronchoalveolar fluids (BALF) from humans or mice. Serum concentrations of MBL, another acute phase reactant with anti-IAV activity [11], [65], [66], are considerably lower at ∼2 µg/ml [14] but can rise up to 3-fold rise during the acute phase response [67]. During infection and inflammation, MBL can enter the airways and BALF levels of 11–78 ng/ml have been reported in patients with pneumonia [68], although the lavage technique will dilute innate immune proteins considerably and levels *in situ* are likely to be significantly higher. By analogy with MBL, it is likely that SAP could enter the lungs during IAV infection at levels that would be sufficient to mediate antiviral activity. Moreover, as SAP potentiates the anti-IAV activity of both SP-D and MBL its presence in IAV-inflamed airway fluids could be an important factor contributing to their ability to promote virus clearance and resolution.

Therapeutic treatment of mice with human SAP has been shown to attenuate fibrotic lung disease [69] and fungal spore-induced allergic airway disease [70]. However, limitations associated with the mouse model of IAV infection have made it difficult to clearly define the *in vivo* role of SAP during IAV infections. For example, mice are not naturally infected with IAV and passage of human isolates through mouse lung selects for mutants with greater replication efficiency [71]. As such, mouse-adapted IAV generally bind α(2,3)-linked SA, the predominant linkage expressed in the murine respiratory tract [72] compared to human strains which display HA receptor preference for α(2,6)-linked SA which is widely expressed by epithelial cells in the upper human airways [73]. Not surprisingly, mice deficient in murine SAP but transgenic for human SAP did not show enhanced susceptibility to PR8 [74], as this mouse-adapted strain displays clear HA preference for α(2,3)-linked SA [75], [76], [77] and is therefore resistant to human SAP (data not shown). In future studies, we propose to utilize the ferret model of IAV infection to assess the therapeutic potential of SAP given that ferrets express abundant α(2,6)-linked SA throughout the respiratory tract [78] and human IAV strains infect ferrets without the need for prior adaptation. Such studies would provide important insight regarding the ability of SAP to ameliorate influenza disease severity and/or virus transmission in a model that more accurately depicts many characteristics of human disease.

## Supporting Information

Figure S1 **IAV grown in human cells remains sensitive to neutralization by human SAP.** IAV virus propagated in human airway epithelial cells is neutralized by SAP. Ud/72 grown in embryonated hens’ eggs (black squares) or in BEAS-2B cell line (white squares) were compared for sensitivity to human SAP using fluorescent focus reduction assay as described in Material and Methods. Results are expressed as a percent of the number of fluorescent foci in the virus only control and data are representative of two independent experiments.
(TIFF)

Table S1 **Mannose-rich glycans on IAV are not the target for recognition of virus by SAP.**
(DOC)

Table S2 **Amino acid at position 226 in the HA sequence from IAV strains of the H3 subtype (1968–1990).**
(DOC)
