# Supplementary figures and images for: Serum Amyloid P Is a Sialylated Glycoprotein Inhibitor of Influenza A Viruses
Source: PLoS One. 2013 Mar 27;8(3):e59623. doi: 10.1371/journal.pone.0059623 (PMC3609861; doi:10.1371/journal.pone.0059623)

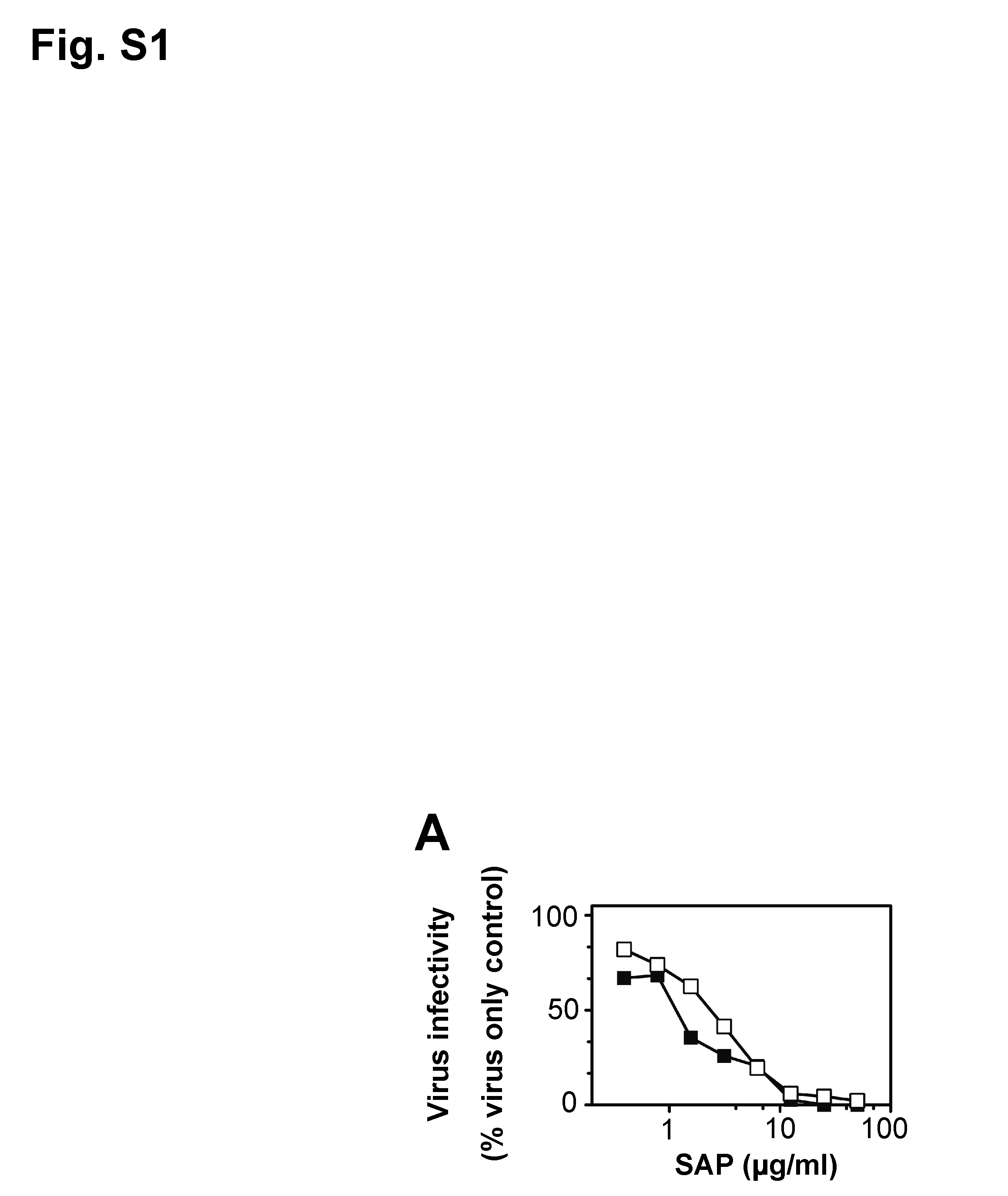

Supplement: Figure S1 — IAV grown in human cells remains sensitive to neutralization by human SAP. IAV virus propagated in human airway epithelial cells is neutralized by SAP. Ud/72 grown in embryonated hens’ eggs (black squares) or in BEAS-2B cell line (white squares) were compared for sensitivity to human SAP using fluorescent focus reduction assay as described in Material and Methods. Results are expressed as a percent of the number of fluorescent foci in the virus only control and data are representative of two independent experiments. (TIFF) [file pone.0059623.s001.tiff]
