# Supplementary material for: Serum Amyloid P Is a Sialylated Glycoprotein Inhibitor of Influenza A Viruses
Source: PLoS One. 2013 Mar 27;8(3):e59623. doi: 10.1371/journal.pone.0059623 (PMC3609861; doi:10.1371/journal.pone.0059623)
Supplement: Table S1 — Mannose-rich glycans on IAV are not the target for recognition of virus by SAP. (DOC) [file pone.0059623.s002.doc]

**Table S1: Mannose-rich glycans on IAV are not the target for recognition of IAV by SAP.**

| **Exp.** | **Virus** | **Treatment of virus** | **HI Titre***^a^* | **MIC***^b^* |
| --- | --- | --- | --- | --- |
|  |  |  | **Bovine serum** | **SAP** |
| **1** | Phil/82 | None | 20480 | 0.625 |
|  | Phil/82-β^R^ | None | 20* | 1.25 |
|  | Mem/71-Bel | None | 5120 | 0.312 |
|  | Mem/71-Bel-β^R^ | None | 80* | 0.312 |
|  |  |  |  |  |
| **2** *^c^* | HKx31 | None | 2560 | 0.312 |
|  |  | Periodate | 80# | 0.625 |
|  |  | Mock - Periodate | 2560 | 0.625 |

*^a^* HI titre is the reciprocal of the highest dilution of bovine serum to inhibit 4 HAU of virus. Titres are representative of two independent experiments.

*^b^* MIC represents the minimum concentration of SAP in μg/ml to completely inhibit 4 HAU of virus. Titres are representative of two independent experiments.

*^c^* Periodate treatment had no effect on ability of virus to agglutinate cRBCs in standard hemagglutination assay (data not shown). For mock-treated virus, periodate was inactivated with glycerol prior to incubation with virus.

* ≥ 4-fold difference in HI titre between wild type and β-inhibitor-resistant virus.

# ≥ 4-fold difference in MIC relative to mock-treated control.
