# Supplementary material for: Serum Amyloid P Is a Sialylated Glycoprotein Inhibitor of Influenza A Viruses
Source: PLoS One. 2013 Mar 27;8(3):e59623. doi: 10.1371/journal.pone.0059623 (PMC3609861; doi:10.1371/journal.pone.0059623)
Supplement: Table S2 — Amino acid at position 226 in the HA sequence from IAV strains of the H3 subtype (1968–1990). (DOC) [file pone.0059623.s003.doc]

**Table S2: Amino acid at position 226 in the HA sequence from IAV strains of the H3 subtype (1968-1990).**

| **Virus strain (H3N2)** | **GeneBank accession** | **Amino acid**  **226** |
| --- | --- | --- |
| A/Aichi/2/1968 | AA43178 | Leu |
| A/Northern Territory/60/1968 | ABF83447 | Leu |
| A/Albany/10/1968 | ABP49514 | Leu |
| A/England/878/1969 | AAA43184 | Leu |
| A/Queensland/7/1970 | AAA43195 | Gln |
| A/Bilthoven/2668/1970 | AAT64670 | Leu |
| A/Memphis/101/1972 | ABD17323 | Leu |
| A/Guadong/243/1972 | ABC67565 | Leu |
| A/Hong Kong/11/1973 | ABB04338 | Leu |
| A/Port Chalmers/1/1973 | ABE12532 | Leu |
| A/Memphis/101/1974 | ABB96352 | Leu |
| A/Albany/42/1975 | ABO52313 | Leu |
| A/Victoria/3/1975 | CAA24270 | Leu |
| A/Memphis/103/1975 | ABC40544 | Leu |
| A/Amsterdam/1609/1977 | AAT64720 | Leu |
| A/Texas/11/1977 | AAL62329 | Leu |
| A/Memphis/12/1978 | ABB96330 | Leu |
| A/Bangkok/1/1979 | ABF21268 | Leu |
| A/Nanjing/13/1980 | ABB53751 | Leu |
| A/Hong Kong/1/1982 | ABB46403 | Leu |
| A/Philippines/2/1982 | AAA18781 | Leu |
| A/Nanjing/28/1984 | ABC86148 | Leu |
| A/Cottbus/42/1988 | CAC81017 | Leu |
| A/Memphis/1/1990 | ABA43336 | Leu |
